# Analysis of Extra Virgin Olive Oils from Two Italian Regions by Means of Proton Nuclear Magnetic Resonance Relaxation and Relaxometry Measurements

**DOI:** 10.1021/acs.jafc.1c00622

**Published:** 2021-04-13

**Authors:** Anton Gradišek, Mario Cifelli, Donatella Ancora, Ana Sepe, Boštjan Zalar, Tomaž Apih, Valentina Domenici

**Affiliations:** † Department of Condensed Matter Physics, Jožef Stefan Institute, 39 Jamova Cesta, SI-1000 Ljubljana, Slovenia; ‡ Dipartimento di Chimica e Chimica Industriale, Università di Pisa, Via Moruzzi 3, 56124 Pisa, Italy

**Keywords:** ^1^H NMR relaxometry, *T*_1_, *T*_2_, lipids, molecular dynamics, olive oil, EVOO

## Abstract

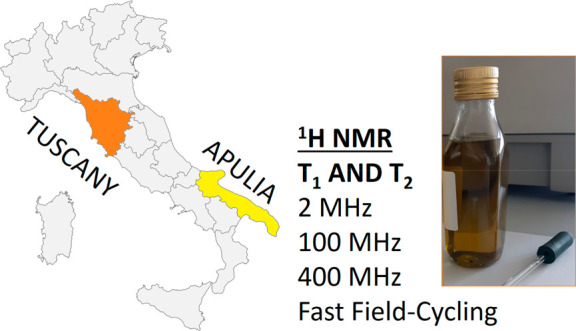

The interest in development of new non-destructive methods for characterization of extra virgin olive oils (EVOOs) has been increasing in the recent years. Among different experimental techniques, nuclear magnetic resonance (NMR) relaxation measurements are very promising in the field of food characterization and authentication. In this study, we focused on relaxation times *T*_1_ and *T*_2_ measured at different magnetic field strengths (namely, 2, 100, and 400 MHz) and ^1^H NMR *T*_1_ relaxometry dispersions directly on olive oil samples without any chemical/physical treatments. A large set of EVOO samples produced in two regions of Italy, Tuscany and Apulia, were investigated by means of ^1^H NMR relaxation techniques. The relaxation studies reported here show several common features between the two sets of EVOO samples, thus indicating that relaxation properties, namely, the ranges of values of *T*_1_ and *T*_2_ at 2 and 100 MHz, are characteristic of EVOOs, independently from the cultivars, climate, and geographic origin. This is a promising result in view of quality control and monitoring.

## Introduction

With the growing awareness of food safety and quality, consumers continuously demand reassurance on food origins and content, and this also applies to extra virgin olive oil (EVOO), an important basis of the Mediterranean diet. There is a great interest by both consumers and producers to have available rapid, cheaper, reliable, and non-destructive screening techniques for the determination of olive oil authenticity at any point of the production and distribution chains.^1−5^ Analytical protocols using high-resolution nuclear magnetic resonance (NMR) spectroscopy^6−8^ have been used in several studies, mainly for the characterization of the triacylglycerol (TAG) fraction of olive oil. ^1^H NMR spectroscopy appears to be the preferred NMR method^9,10^ as a result of its higher sensitivity and shorter relaxation times of proton nuclei with respect to other nuclei, such as carbon 13^11−13^ and phosphorus 31.^14,15^ However, some limitations exist also in the case of ^1^H NMR; i.e., the presence of scalar coupling between the neighboring protons and the much smaller chemical shift ranges (∼15 ppm) for protons often result in overcrowded spectra with severe signal overlap, making the analysis of the spectra more difficult.^8^ The ^1^H NMR spectra of olive oil samples consist of 10 major signals attributed to the fatty acyl chains and the glyceryl protons of TAGs. Even if ^1^H NMR analysis is not able to show the positional distribution of fatty acids on the glycerol backbone, the combination with ^13^C NMR spectroscopy analysis can help in assigning all signals. In this way, it is possible to have a lot of information on the saponifiable fraction of olive oils. On the contrary, most of the numerous minor compounds of the unsaponifiable fraction cannot be easily quantified by ^1^H NMR, except in specific cases.^16−18^ Recent studies have been published, in which NMR has been used for the prediction of the olive oil geographical origin using NMR combined with multivariate statistical methods^19^ and to discriminate olive oils obtained from olives produced in different pedoclimatic conditions in combination with other spectroscopic techniques.^20^

Only a small number of studies about ^1^H NMR relaxation measurements are present in the literature about olive oil and, in particular, about EVOOs.^21,22^ The study of ^1^H NMR relaxation times, longitudinal (*T*_1_) and transverse (*T*_2_), as a function of the temperature and/or at variable Larmor frequencies is an attractive approach to study liquids.^23^ Because the relaxation times depend upon the chemical composition, viscosity, and other chemical–physical properties, the analysis of the relaxation times can help to distinguish among different kinds of oils.^21,24−26^ An approach proposed by Conte et al.^27^ showed the efficiency of the nuclear magnetic resonance relaxation dispersion (NMRD) technique in the evaluation of differences among oils obtained from seeds subjected to different thermal desiccation processes and retrieved from seeds belonging to the same cultivar grown in different geographical areas. In this case, the measurements of ^1^H longitudinal relaxation times (*T*_1_) at different frequencies were applied on pistachio oil samples, to extract parameters, such as the correlation times for molecular motions. Similar approaches were used on several vegetable oil samples.^28,29^ Considering the temperature sensitivity of food systems and the fact that oil is subject to oxidative stresses already at 35 °C, it is difficult to retrieve dynamic information through the temperature dependence of relaxation times.^21^ Therefore, instead of temperature-dependent measurements, the evaluation of the relaxation rates at a constant temperature but variable Larmor frequencies was applied, by means of the ^1^H NMRD technique, also known as fast field cycling (FFC) relaxometry. Furthermore, low-field NMR setups that use permanent magnets are being used in a variety of applications that do not require high-resolution spectra but instead only focus on spin relaxation. Portable NMR instruments working with a static low magnetic field have several advantages, such as the relatively low cost of equipment. Moreover, they do not rely on cryogens; they allow for the performance of fast measurements; and the measurement setup is relatively easy to manage. On the other hand, the interpretation of the results may not be so straightforward, and in some cases, it requires statistical analysis or advanced knowledge of data modeling.

In this paper, we explore the possibility of characterization of EVOOs using a variety of ^1^H NMR relaxation techniques, namely, proton spin relaxation *T*_1_ and *T*_2_ measured at the magnetic fields of 2, 100, and 400 MHz, which were coupled to the measurements of *T*_1_ dispersions acquired by FFC NMR relaxometry. With this aim, a large set of EVOOs produced in two regions of Italy, namely, Tuscany and Apulia, was studied, and the common features obtained in the relaxation data are finally discussed in terms of possible applications for EVOO quality control.

## Materials and Methods

### EVOO Samples

The EVOO samples analyzed in this work were provided by olive oil producers and local farms in Tuscany (32 EVOO samples) and Apulia (35 EVOO samples), as reported in Table S1 of the Supporting Information. For simplicity, the EVOO samples are labeled as “at_*X*” and “ap_*Y*” to indicate whether they are from Tuscany or Apulia, respectively, where *X* and *Y* are consecutive numbers to identify the EVOO samples. EVOO samples were characterized by means of standard techniques to evaluate the specific olive oil category directly by the producers [for instance, oil acidity and ultraviolet (UV) parameters K232 and K270]. When not used in experiments, all oil samples were stored in dark conditions, in 25 mL dark glass bottles, at a temperature of ≤5 °C. Among the samples, the at_28 EVOO was chosen for a detailed analysis because it exhibited all typical EVOO features by means of physical and chemical characterization^30^ and sensorial tests; furthermore, it was available in a sufficient quantity to perform several different measurements to ensure the reproducibility of all NMR measurements.

### NMR Instruments

^1^H NMR relaxation measurements on oil samples were performed using different NMR spectrometers working at ^1^H Larmor frequencies of 2, 100, and 400 MHz and by a FFC setup in a range from 10 kHz to 10 MHz. In the following, the technical features of the NMR instruments are briefly described.

A rock core analyzer spectrometer (Magritek, http://www.magritek.com/) operating at ^1^H Larmor frequency of 2 MHz was used to determine the proton spin–lattice relaxation times, *T*_1_, and proton spin–spin relaxation times, *T*_2_. This instrument is a wide-bore NMR system, using a permanent magnet, typically working at low resolution, specifically for soft and solid matter (it was originally developed to measure porosity of concrete or the oil content in rocks). About 20 mL of oils was transferred to weighing bottles (Ø = 30 mm and *V* = 20 mL) and put in the large bore at room temperature with a temperature control of ±0.5 °C. The inversion recovery sequence was used for *T*_1_ measurements, with variable time delay τ from 1 ms to 1 s in 20 steps and a 90° pulse of 20 μs. The number of scans was 4 per sequence, and the delay time between the repetitions was equal to 3 s. The Carr–Purcell–Meiboom–Gill (CPMG) sequence^31,32^ was used for *T*_2_ measurements. The time delay τ was 200 μs, and 1000 echos were used. The 90° pulse was 40 μs. The number of scans was 16, and the repetition time was 0.5 s.

Measurements of ^1^H NMR relaxation times *T*_1_ and *T*_2_ were performed using a horizontal bore Oxford magnet operating at 100 MHz. The temperature was controlled by a gas flow system, and the temperature control was ±0.5 °C. About 2 mL of oil was transferred to MRI glass tubes (Ø = 0.5 cm and *h* = 1.5 cm) and put into the probe. For the inversion recovery sequence used for *T*_1_ measurements, the 90° pulse was 3.5 μs and the time delay τ varied from 0.2 ms to 3 s in 21 steps. The number of scans was 2, and the delay time was equal to 3 s. The spin echo sequence was used for *T*_2_ measurements. The time delay τ was varied from 0.02 ms to 2 s in 12 steps; the 90° pulse was 3.5 μs; and the number of repetitions was 2, with a delay time equal to 3 s. A temperature control of ±0.1 °C was used.

^1^H NMR relaxation times (*T*_1_) for different signals of the ^1^H NMR spectra were measured using a Bruker DRX Advance 400 MHz NMR spectrometer using the inversion recovery sequence. The time delay τ varied from 1 ms to 10 s in 16 steps; the 90° pulse was 14 μs; the number of scans was 16; and the delay time was equal to 10 s.

^1^H NMR dispersion of *T*_1_ data was acquired using the FFC NMR relaxometer SPINMASTER FFC 2000 (Stelar srl). The *T*_1_ values were measured in the frequency range from 10 MHz to 10 kHz at 21.0 ± 0.5 °C. The temperature was controlled using a standard gas flow system. For frequencies higher than 8 MHz, a non-prepolarized pulse sequence (NPS) was used, while below that frequency, *T*_1_ values were obtained using a prepolarized pulse sequence (PPS). A 6.2 μs proton 90° pulse and a maximum value of the recycle delay of 0.5 s were used. Each *T*_1_ measurement was performed in 25 blocks of 4 ms. The probe dead time was around 40 μs. Other parameters were optimized according to each measurement.

### Spectral and Data Analysis

NMR data were analyzed using integration of the spectra. In the case of the high-resolution ^1^H NMR spectra (400 and 100 MHz), parts of the spectra were integrated to obtain the mono-exponential spin–lattice or spin–spin relaxation rates. In the case of the low-resolution spectra (2 MHz and FFC), the entire broad spectra were integrated and the relaxation times were obtained using a two-component relaxation decay. Relaxometry data were analyzed using a homemade software package in the MATLAB environment. Fitting of the ^1^H NMR *T*_1_ dispersions in terms of dynamic models was carried out using a nonlinear least squares minimization with the Fitteia software.^33^ The validation and reproducibility of the ^1^H NMR relaxation measurements were tested on the reference EVOO sample, namely, at_28, by doing measurements in triplicate and calculating the average values of *T*_1_ and *T*_2_ at different Larmor frequencies and relative error. In the case of *T*_1_ measured at 2 MHz, the error found was about 2% (component “a”) and 5% (component “b”), and at 100 MHz, the error is in the range of 1–2%, while at 400 MHz, the error on the different relaxation times measured for the different ^1^H signals is less than 3%. In the case of *T*_2_, the relative error at 100 MHz ranges between 2 and 8%, while the error found at 2 MHz in component “a” of *T*_2_ is 1% and in the component “b” of *T*_2_ is 5%. In the case of ^1^H NMR relaxometry, several runs of *T*_1_ dispersion were performed on the EVOO sample at_28, and we obtained almost perfectly reproducible trends.

## Results and Discussion

### ^1^H NMR Spectra of EVOOs

**Figure 1:**
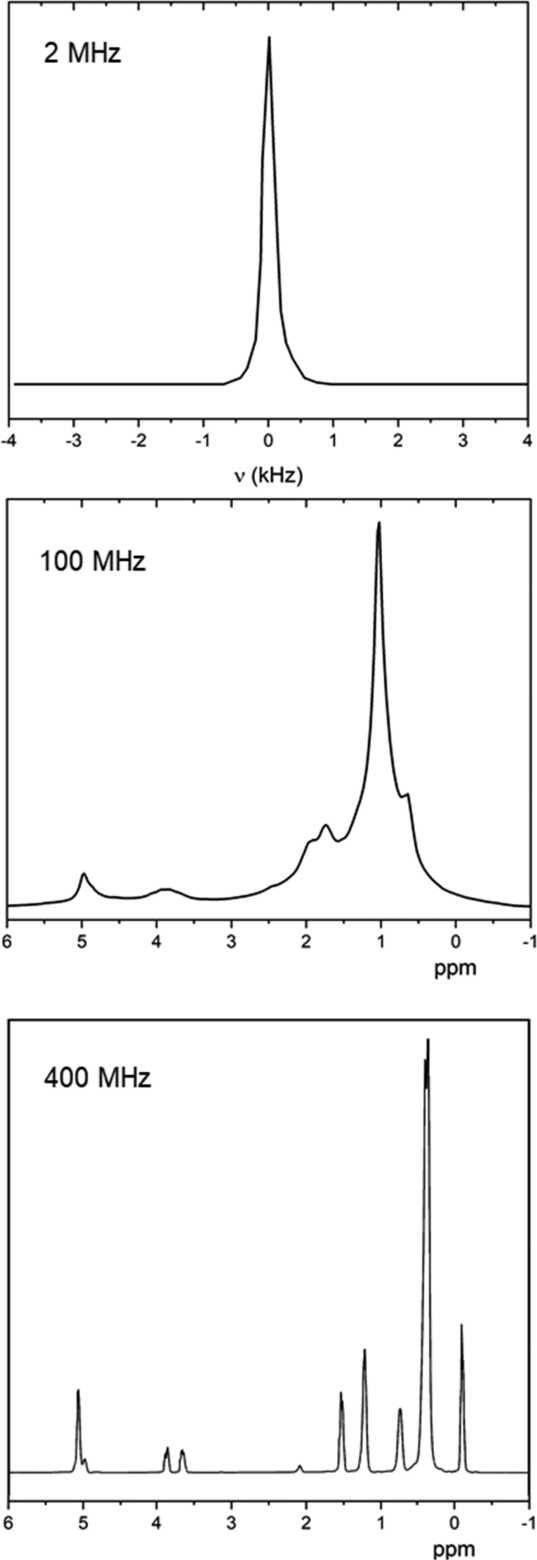
^1^H NMR spectra of a representative EVOO sample (at_28) recorded at 2 MHz permanent magnet and at 100 and 400 MHz superconducting magnets at room temperature.

Figure 1 shows the proton NMR spectra of a representative EVOO sample from Tuscany (at_28) recorded at room temperature at Larmor frequencies of 2, 100, and 400 MHz. The 2 MHz permanent magnet has a low magnetic field homogeneity; therefore, the spectrum is seen as a single line, a roughly Lorentzian shape with full width at half maximum of ∼0.4 kHz (top image of Figure 1). The proton spectra obtained at the FFC setup are similarly featureless (not shown here), but it should be noted that this setup is not designed for high-resolution NMR spectroscopy.

The ^1^H NMR spectra at 100 and 400 MHz (middle and bottom images of Figure 1) consist of several peaks in the region between 0 and 5.5 ppm. They correspond to chemically different proton groups referred to as the fatty component, which represents about 98% of all chemical components of EVOOs.^6,8^ The spectrum recorded at 100 MHz, because the operating magnet has a lower field homogeneity, shows three regions with broad and partially overlapped signals. In particular, the two broad and less intense signals centered at 5 and 3.8 ppm correspond to CH present in unsatured fatty acids and triacylglycerols and to CH_2_ present in triacylglycerols, respectively. Moreover, the most intense peak between 0 and 2.2 ppm is due to the superposition among several signals, mainly as a result of CH_2_ of acyl chains and unsatured fatty acids and a minor contribution as a result of CH_3_ present in terminal acyl chains of fatty acids. The high-resolution ^1^H NMR spectrum recorded at 400 MHz has the typical features already widely investigated and reported in the literature.^6,8^ Here, 10 separate ^1^H signals can be assigned (see Table 1). As seen in Figure 1 and reported in Table 1, the largest contribution to the ^1^H NMR spectrum is related to CH_2_ protons, while the smallest contribution is due to CH (chemical shift larger than 5 ppm), and a relatively intense signal with a chemical shift lower than 1 ppm is due to the CH_3_ group.^8^

**Table 1:** Chemical Shifts (δ in ppm) and Assignment of the Signals in the ^1^H NMR Spectrum of an EVOO Sample Recorded at 400 MHz

δ (ppm)	proton group	attribution to the EVOO fatty component
5.5–5.2	–C**H**=CH–	all unsatured fatty acids
5.1	C**H**–OCOR	triacylglycerols
4.3–4.0	C**H**_**2**_–OCOR	triacylglycerols
2.7	CH=CHC**H**_**2**_CH=CH	lynoleic and lynolenic chains
2.3	C**H**_**2**_–COOH	all acyl chains
2.0	C**H**_**2**_CH=CH	all unsatured fatty acids
1.6	C**H**_**2**_CH_2_–COOH	all acyl chains
1.2	–(C**H**_**2**_)_*n*_–	all acyl chains
0.9	CH=CH–CH_2_–C**H**_**3**_–	lynolenic acid
0.8	CH_2_CH_2_CH_2_–C**H**_**3**_–	all acyl chains exept lynolenyl

### ^1^H NMR Spin–Lattice Relaxation

Proton spin–lattice (or longitudinal) relaxation is the process in which the nuclear magnetization recovers to the equilibrium value along the direction parallel to the static magnetic field. In the simplest situation, magnetization recovery can be described by an exponential function with a characteristic constant called the spin–lattice relaxation time, *T*_1_. Spin–lattice relaxation is influenced by fluctuations in the dipolar interaction between proton spins. In non-confined liquids without paramagnetic components, these fluctuations are typically caused by molecular motions, such as molecular rotations/reorientations and self-diffusion. However, the mobility of different proton group varies, thus influencing their relaxation. For example, the protons in a CH_3_ group at the end of a chain can rotate fast around the C–C axis, while the CH_2_ protons in the middle of the chain will have a reduced mobility. Protons around double or conjugated bonds or on benzene rings have less mobility, and their motion can be tied to the rest of the molecule. Such effects were well-studied in liquid crystals.^34,35^ As the CH_3_ protons rotate fast, they will see a more substantial averaging out of the fluctuations in the dipolar interaction than the more rigid protons; thus, *T*_1_ for the CH_3_ protons may be longer. This is an effect often observed in long molecules (such as in lyotropic liquid crystals or other liquid crystalline systems), while in shorter molecules, spin diffusion causes all proton spins to relax at the same rate.^36,37^

When dealing with the high-resolution ^1^H NMR spectra, such as in our case at 100 and 400 MHz, spin–lattice relaxation times for particular proton groups can be obtained by integrating the corresponding parts of the spectra. Table 2 shows the values of *T*_1_ corresponding to different regions of the spectra, as described in the previous section, for the reference EVOO sample (at_28). The values of *T*_2_ are added for 100 MHz, as discussed in the following.

**Table 2:** Proton NMR Spin–Lattice Relaxation Times for Different Proton Groups Measured on the Reference EVOO Sample (at_28) at Room Temperature^a^

peak (chemical shift, ppm)	5.5–5.2	5.1	4.3–4.0	2.7	2.3	2.0	1.6	1.2	0.9	0.8
*T*_1_ (ms) at 100 MHz^b^	295 ± 6		122 ± 2	191 ± 3				235 ± 4	352 ± 6	
*T*_1_ (ms) at 400 MHz	873 ± 22	493 ± 14	413 ± 11	445 ± 10	437 ± 13	504 ± 11	462 ± 12	466 ± 10	656 ± 15	740 ± 12

a All data are expressed as the average value ± standard deviation measured in triplicate.

b Note that several peaks overlap at 100 MHz; therefore, some values of *T*_1_ are the same.

On the other hand, when dealing with a setup with a low field inhomogeneity, the spectral lines merge to a single broad line, which does not allow us to use the same approach as above. Instead, spin–lattice relaxation times are determined by integrating the entire spectra.

**Figure 2:**
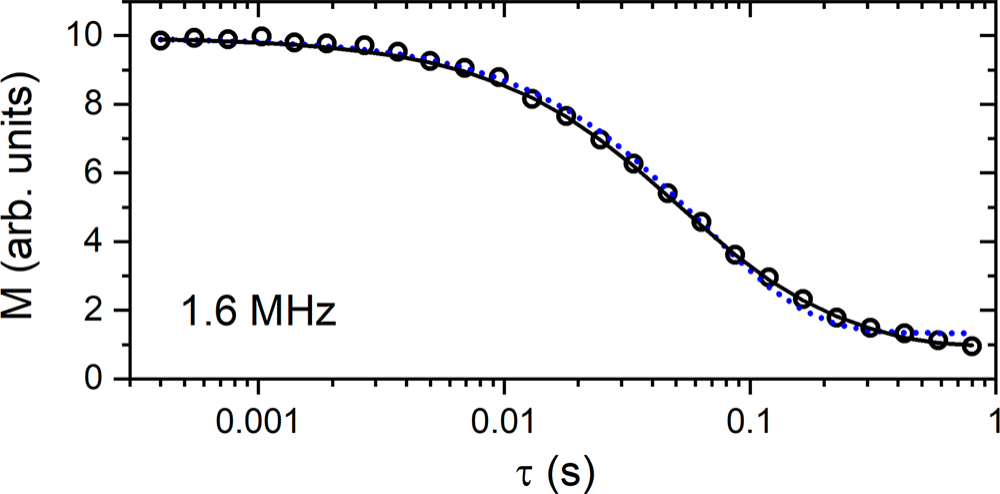
Magnetization decay (circles) of a representative EVOO sample (at_28) measured at 1.6 MHz at the FFC setup with the prepolarized sequence at room temperature. The solid black line represents the two-component relaxation model fit, while the blue dash-dot line represents the best fit using a mono-exponential model. The parameters for both models are listed in the Supporting Information.

Figure 2 shows the magnetization decay curve for the reference EVOO sample (at_28) at room temperature, measured at the FFC setup. From the figure, it is clear that the relaxation is not mono-exponential (blue dash-dot line), but it is instead better described using a sum of two exponential functions (solid black line). Because EVOOs consist mainly of triglycerides, the two components in relaxation likely belong to different groups of protons within the triglyceride molecules. In the analysis of multicomponent relaxation data in liquids consisting of a single type of molecule, it is possible to assign the weights to the two components proportional to the number of each type of protons in the molecule. However, in EVOOs, there are many different types of triglycerides, with different fatty acids, such as oleic, linoleic, palmitic, and other acids, with other chemical compounds present in traces. Therefore, in the analysis of the relaxation data for EVOOs, we let the amplitude ratio as a free parameter and the weights of components obtained were typically around 2:1 for the short component. The component with the shorter *T*_1_ is attributed to the more rigid protons on the molecular chains (for instance, the CH protons in the unsaturated fatty acids, CH and CH_2_ in the glycerol unit, and CH_2_ in the fatty acids closer to the glycerol unit), while the component with the longer relaxation time is attributed to the more mobile parts of the molecule (for instance, CH_3_ and CH_2_ closer to the terminal chains of the fatty acids). The same effect was observed in the analysis of the magnetization recovery measurements on the 2 MHz setup (not shown here), with two clear components of relaxation with comparable amplitudes.

**Figure 3:**
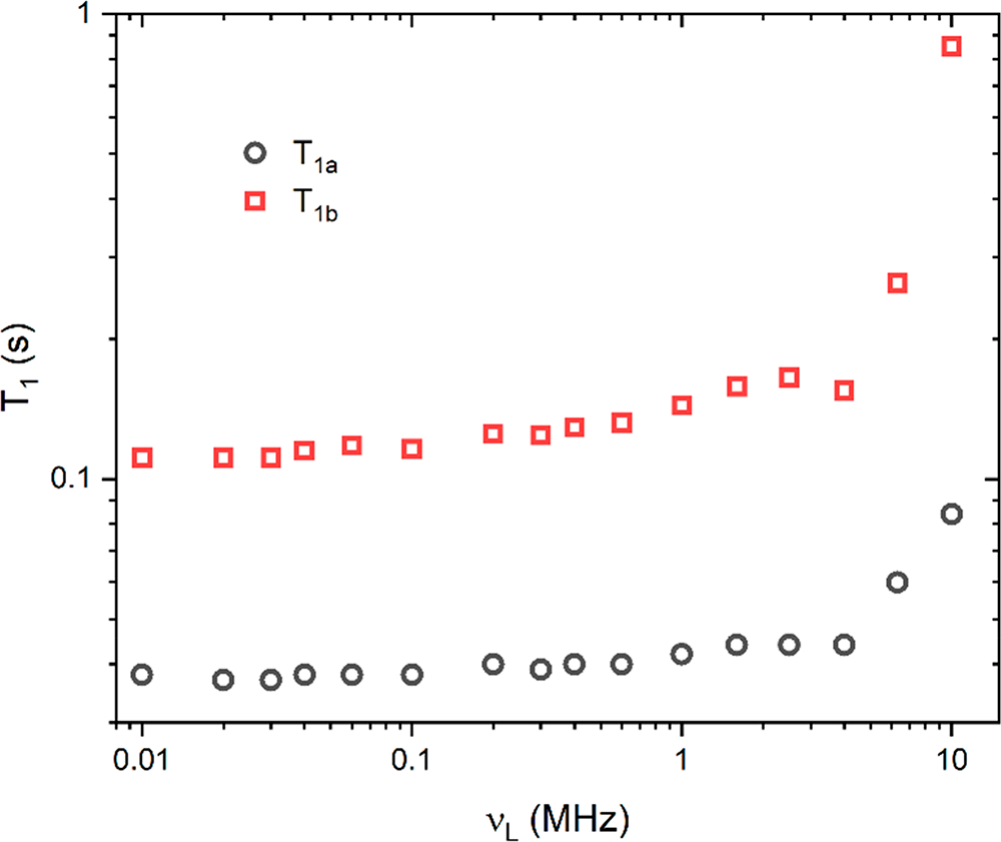
Proton spin–lattice relaxation of a representative EVOO sample (at_28) measured at FFC, using a two-component analysis of the magnetization relaxation curves, as described in the text.

Figure 3 shows the ^1^H NMR *T*_1_ relaxation dispersion for the reference EVOO sample (at_28) at room temperature, measured in the proton Larmor frequency range from 10 kHz to 10 MHz. Both components show similar behavior at low frequencies, and the relaxation profiles are flat, while *T*_1_ starts increasing at higher frequencies.

To analyze the field dependence of the relaxation data, we will focus here on the component with the shorter *T*_1_, which corresponds to the protons in the more rigid part of the molecules. To obtain a wider frequency range, we supplement the FFC data with the values of *T*_1_ measured at 100 and 400 MHz, where we use the average values for the CH and CH_2_ proton signals. In the analysis, we consider two dynamic processes that influence the proton relaxation: molecular rotations/reorientations and molecular self-diffusion.^27^

In this approach, the contributions of the different motions to the relaxation rate are considered additive,^33^ so that we can compute the total relaxation rate of the system as a sum of different contributions.

The simplest model to describe the relaxation contribution as a result of molecular rotations/reorientations is the Bloembergen–Purcell–Pound (BPP) model^38^
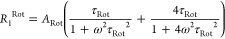
1where *R*_1_ = 1/*T*_1_ is the relaxation rate, ω = 2π*v*_L_, *A*_Rot_ is the prefactor, and τ_Rot_ is the rotational correlation time. The latter has an Arrhenius-like temperature dependence; however, because we are analyzing the data only at room temperatures, we will consider *A*_Rot_ and τ_Rot_ as constant values.

To describe the contribution of the molecular self-diffusion to the relaxation, we use the model developed by Torrey.^39^ This is a phenomenological model specifically adapted for lyotropic and other liquid crystalline materials, where the relaxation rate (*R*_1_) depends upon the self-diffusion constant, *D*. In our analysis, we have used the value experimentally determined using the diffusion ordered spectroscopy (DOSY) ^1^H NMR experiment^40^ on the reference EVOO sample (at_28): *D* = 7.7 ± 0.5 × 10^–12^ m^2^/s.

**Figure 4:**
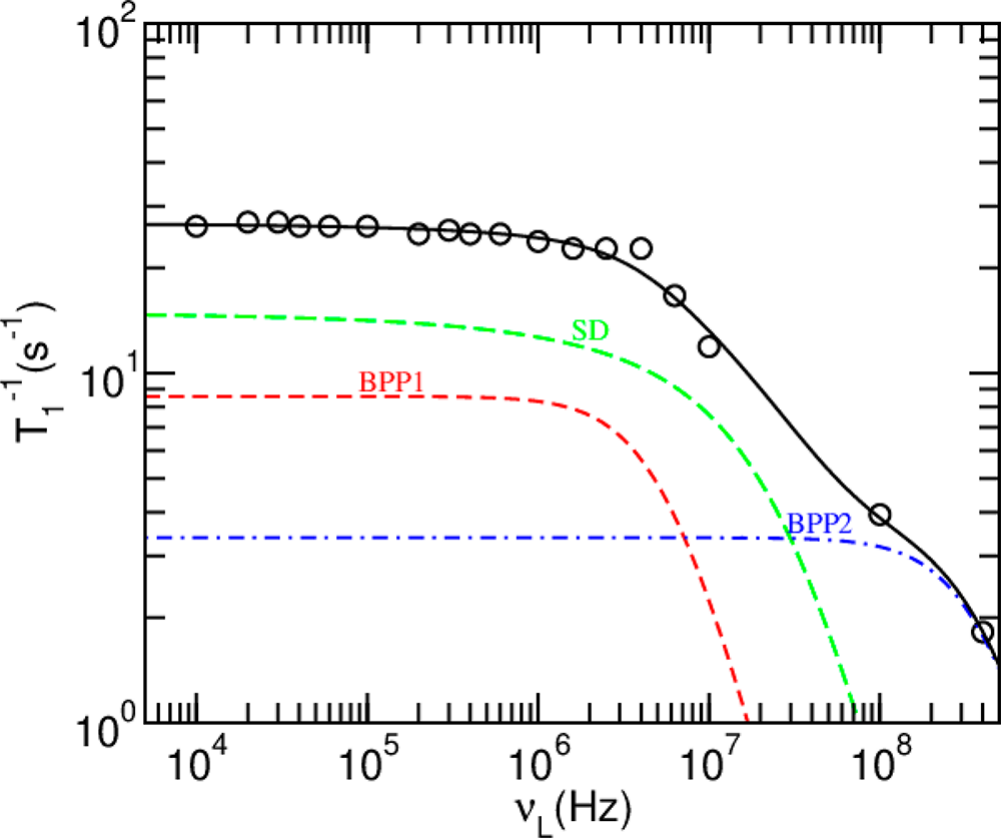
Spin–lattice relaxation rate (circled) as a function of the proton Larmor frequency. Contributions of individual mechanisms (BPP1, BPP2, and SD) are shown together with the sum of the contributions (solid black line).

From the analysis of the experimental data using the above relaxation models, it is clear that the data cannot be fully explained using solely a single BPP contribution and the self-diffusion (SD) contribution. Discrepancies between experimental and computed relaxation rates appear at the highest frequencies (above 10 MHz). Instead, we are required to consider an additional BPP contribution. In the analysis of rod-like molecules, the two rotational mechanisms can be imagined as rotations along the long and short molecular axes, and each of them has a separate correlation time, *τ*_c_. Because EVOOs are complex mixtures of (albeit mostly similar) compounds, structure-specific parameters can only be approximated. Considering this limitation, we can assume that the BPP contribution relevant at high frequencies could represent a fast reorientation along the main longitudinal axis of the triglyceride molecules, while the BPP contribution active at lower frequencies could take into account both tumbling reorientations of the whole triglyceride and the single fatty acids. A reasonable fit of the relaxation data is shown in Figure 4, and it is obtained using two BPP contributions (BPP1 and BPP2) and the SD motion, which is fixed from independent measurements. For the BPP1 mechanism, we obtained the parameters *A*_Rot1_ = 1.08 × 10^8^ s^–2^ and τ_1_ = 1.5 × 10^–8^ s, while for the BPP2 mechanism, we obtained *A*_Rot2_ = 3.15 × 10^9^ s^–2^ and τ_2_ = 2.15 × 10^–10^ s. As stated above, the (known) value of the diffusion constant has been fixed.

### ^1^H NMR Spin–Spin Relaxation

Proton spin–spin (or transversal) NMR relaxation is a process where the detectable transverse component of the nuclear magnetization looses coherence, finally reducing to zero, and it is characterized by the relaxation time *T*_2_. Similar to *T*_1_, the process is typically exponential in homogeneous systems. As opposed to spin–lattice relaxation, which is governed by the fluctuations of dipolar interactions close to the Larmor frequency, spin–spin relaxation is affected by the fluctuations at low frequencies. In the simplest picture, *T*_2_ should be independent of the external magnetic field.

In our study, we measured *T*_2_ with two NMR instruments working at 100 and 2 MHz. At 100 MHz, the resolved proton NMR spectra allow us to determine the *T*_2_ values for different proton species. Each of the lines show exponential decay. The *T*_2_ values for the reference EVOO sample (at_28) are 40 ± 3 ms for the signal between 5.1 and 5.5 ppm, 22 ± 2 ms for the signal centered at 4.3–4.1 ppm, 33 ± 2 ms for the signal between 1.6 and 2.7 ppm, 56 ± 4 ms for the most intense signal centered at 1.2 ppm, and 47 ± 3 ms for the small signal below 1 ppm (see Figure 1 for the ^1^H NMR spectrum at 100 MHz).

On the other hand, as a result of the broad proton line of the NMR spectrum recorded at 2 MHz, spin–spin relaxation has to be analyzed using the entire spectra. In line with the *T*_1_ measurements reported in the previous section, spin–spin relaxation is also not mono-exponential and can be fitted taking into account two relaxation components with magnitudes similar in size. Again, as before, we let the amplitudes as free fitting parameters. For the reference EVOO sample (at_28), the two obtained components are 43 ± 5 and 147 ± 12 ms, with the shorter value being close to the values obtained at 100 MHz.

As discussed in the following section, spin–lattice and spin–spin relaxation times at 2 and 100 MHz have been measured for a large set of EVOO samples produced in Italy.

### Relaxation Times Measured on a Large Set of EVOOs Produced in Two Italian Regions: Apulia and Tuscany

Up to this point, we have reported relaxation data recorded on a reference EVOO sample (“at_28”) at different magnetic fields and with different methods to obtain spin–spin and spin–lattice ^1^H NMR relaxation times. Among the NMR techniques explored in this work, not all of them are appropriate for fast screenings of a large number of samples. As reported in the previous sections, the use of FFC ^1^H NMR relaxometry allows us to extract a wealth of information about the molecular dynamics of the EVOO sample. However, measuring the entire ^1^H NMR relaxation dispersion is time-consuming and needs to be supplemented by the use of relaxation measurements at high magnetic fields to properly cover the field range, where one of the rotational motions becomes the dominant mechanism contributing to spin–lattice relaxation.

**Figure 5:**
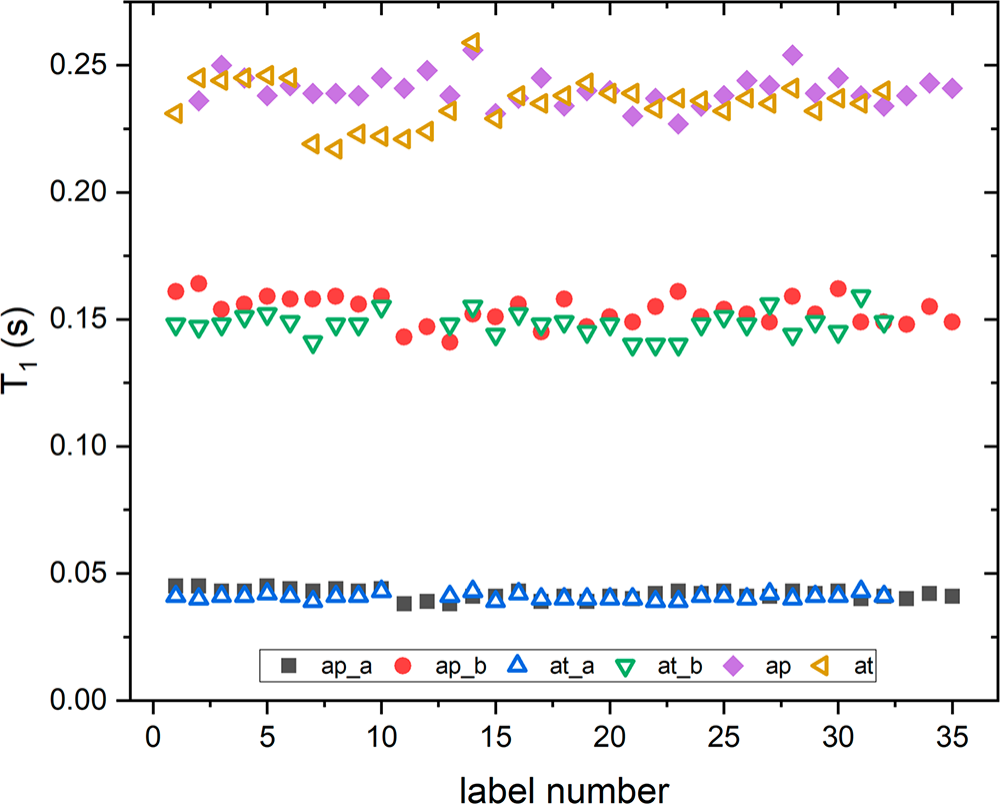
Spin–lattice relaxation times measured on a large set of EVOOs: ap_a and ap_b are the two components of relaxation times measured at 2 MHz for EVOOs from Apulia, and ap is the value of the relaxation time measured at 100 MHz. In the same manner, the label at denotes the EVOOs from Tuscany.

**Figure 6:**
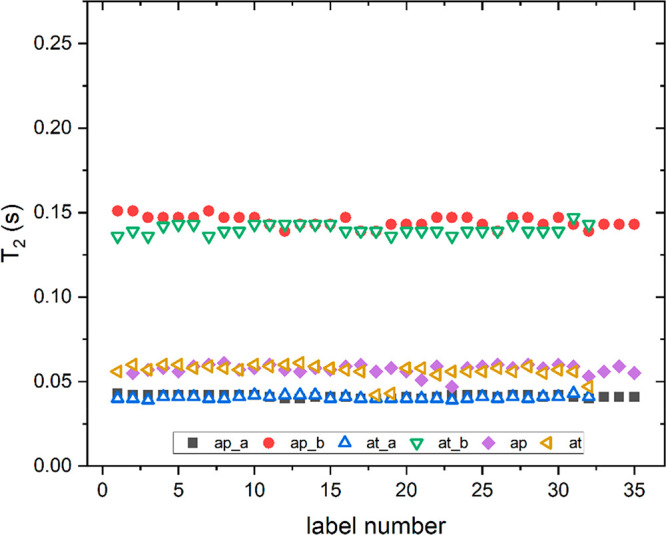
Spin–spin relaxation times measured on a large set of EVOOs: ap_a and ap_b are the two components of relaxation times measured at 2 MHz for EVOOs from Apulia, and ap is the value of the relaxation time measured at 100 MHz. In the same manner, the label at denotes the EVOOs from Tuscany.

On the contrary, here, we argue that the measurements of ^1^H NMR spin–lattice and spin–spin relaxation times at a single magnetic field can be useful for a fast characterization of olive oils as well. Figures 5 and 6 show *T*_1_ and *T*_2_ values for a large set of EVOOs from two different Italian regions, namely, Apulia and Tuscany, at 100 and 2 MHz. At 100 MHz, both values of *T*_1_ and *T*_2_ were obtained by integrating the most intense peak in the proton NMR spectrum (see Figure 1, with a chemical shift from 0 to 2.5 ppm). In both cases, a mono-exponential function is used, which perfectly reproduced the τ dependence of the magnetization. At 2 MHz, the relaxation decay was fitted using a two-component model, which gave rise to two distinct values of relaxation times, named as component “a” and component “b” in Figures 5 and 6. For an easier comparison, the values for *T*_1_ and *T*_2_ are plotted on the same vertical scale.

Almost all EVOO samples were produced from olives harvested in 2012 under similar pedoclimatic conditions. However, it is well-known that Tuscan and Apulian EVOO samples are produced from different olive cultivars: Frantoio, Leccino, and Moraiolo are typical of Tuscany, while Cellina di Nardò, Ogliarola, Coratina, and Cima di Bitonto are more typical of Apulia. Some other differences between the two regions concern the geographical features of Tuscan hills with respect to the more flat area of Apulia. In principle, the two sets of EVOO samples could present some differences, also from the chemical point of view, as revealed by high-resolution ^1^H NMR studies combined with multivariate statistical analysis.^41−43^ However, as easily observed in Figures 5 and 6, the trends of relaxation times at the two magnetic fields are very similar between the two sets of EVOOs. Considering the eventual differences in the spectral features between EVOOs produced in different geographic areas,^41−43^ the fact that the relaxation times, both *T*_1_ and *T*_2_, show very similar values, except for a few statistical oscillations, could be a positive aspect to distinguish EVOO samples from oils of different botanical origins as well as to detect adulterations. As will be reported in a follow-up paper, the relaxation data obtained at 2 and 100 MHz are very sensitive to the type of oil, which is related mostly to the fatty acid constituents.^40^

On the other hand, the present work confirms that the NMR methods based on relaxation measurements at low fields are valuable options to check the authenticity of EVOOs, as reported in previous papers about the use of the time-domain NMR technique.^44,45^ With respect to high-resolution NMR methods based on the spectral analysis,^17,41,42^ the relaxation data analysis reported here does not require the use of multivariate statistical techniques to extract relevant information from the spectra. However, the ability of the present NMR relaxation approach to detect adulterations on EVOOs will be the subject of a separate work.

## Supporting Information Available

Detailed list of EVOO samples (Table S1) and model fits and parameters associated with Figure 2 (PDF)
