# Supplementary material for: Analysis of Extra Virgin Olive Oils from Two Italian Regions by Means of Proton Nuclear Magnetic Resonance Relaxation and Relaxometry Measurements
Source: J Agric Food Chem. 2021 Apr 13;69(41):12073–80. doi: 10.1021/acs.jafc.1c00622 (PMC8532148; doi:10.1021/acs.jafc.1c00622)
Supplement: Supplementary file 1 — jf1c00622_si_001.pdf [file jf1c00622_si_001.pdf]

# Analysis of Extra-Virgin Olive Oils from two Italian Regions by Means of $^1$H NMR Relaxation and Relaxometry Measurements – Supporting information

*Anton Gradišek[1*], Mario Cifelli[2], Donatella Ancora[2§],*

*Ana Sepe[1], Boštjan Zalar[1], Tomaž Apih[1] and Valentina Domenici[2*]*

1. Department of Condensed Matter Physics, Jožef Stefan Institute, 39 Jamova Cesta, SI-1000, Ljubljana (Slovenia);

2. Dipartimento di Chimica e Chimica Industriale, Università di Pisa, via Moruzzi, 3 – 56124 Pisa (Italy).

[§] former student at Dipartimento di Chimica e Chimica Industriale.

* Corresponding authors,

E-mail: anton.gradisek@ijs.si & valentina.domenici@unipi.it

***Table S1**: A detailed list of EVOO samples used in the study.*

| Sample | Producer | Area | Cultivar | Year | Further information | Label for Figures |
|---|---|---|---|---|---|---|
| **at_1** | Agriturismo Pane&Vino | Gabbro (LI) | blend | 2012 | biologic oil | 1 |
| **at_2** | Agriturismo Cappellese | Castelnuovo della Misericordia (LI) | Frantoio, Leccino, Moraiolo | 2012 | biologic oil | 2 |
| **at_3** | Azienda Agrituristica | Larciano (PT) | blend | 2010 | biologic oil | 3 |

| | | | | | | |
|---|---|---|---|---|---|---|
| | Casolar de No' altri | | | | | |
| **at_4** | Azienda Orzalesi | Rosignano Marittimo (LI) | Frantoio, Leccino, Moraiolo | 2012 | | 4 |
| **at_5** | Azienda Agricola "Le Ceppite" | Rosignano Marittimo (LI) | Frantoio, Leccino, Moraiolo | 2012 | | 5 |
| **at_6** | Azienda Agricola "Antica Fonte" | Rosignano Marittimo (LI) | Frantoio, Leccino, Moraiolo | 2012 | | 6 |
| **at_7** | Azienda Agricola "Esposito Susanna et Atria" | Bibbona (LI) | Frantoio, Leccino, Moraiolo | 2012 | | 7 |
| **at_8** | Franci Frantoio IGP | Montenero D'orcia | Frantoio, Leccino, Moraiolo | 2012 | | 8 |
| **at_9** | Oliveto Fonte di Foiano | Castagneto Carducci | Frantoio, Leccino, Moraiolo | 2012 | | 9 |
| **at_10** | Azienda Agricola Giovani | San Lorenzo, Suvereto | Frantoio, Leccino, Moraiolo | 2012 | | 10 |
| **at_11a** | Tenuta "La Pineta" | Catiglion Fibocchi (AR) | Frantoio, Leccino, Moraiolo | 2011 | Unfiltered | 11 |
| **at_11b** | Tenuta "La Pineta" | Catiglion Fibocchi (AR) | Frantoio, Leccino, Moraiolo | 2012 | Filtered oil | 12 |
| **at_12** | Tenuta "S.Jacopo" | Cavriglia (AR) | Frantoio, Leccino, Moraiolo | 2012 | | 13 |
| **at_13** | Azienda Agricola Cristiana Ruschi | Calci (PI) | Frantoio, Leccino, Moraiolo | 2012 | | 14 |
| **at_14** | Azienda Mannucci Doandri DOP | Ceppeto (AR) | blend | 2011 | | 15 |
| **at_15** | Azienda "La Salceta" | Lorociuffenna (AR) | Frantoio, Leccino, Moraiolo | 2012 | | 16 |
| **at_16a** | Tenuta "Montefoscoli " | Palaia (PI) | Frantoio, Leccino, Moraiolo | 2010 | Biologic oil | 17 |
| **at_16b** | Tenuta "Montefoscoli " | Palaia (PI) | Frantoio, Leccino, Moraiolo | 2011 | Biologic oil | 18 |
| **at_16c** | Tenuta "Montefoscoli " | Palaia (PI) | Frantoio, Leccino, Moraiolo | 2012 | Biologic oil | 19 |
| **at_17** | Azienda Società | Siena (SI) | blend | 2011 | | 20 |

| | | | | | | |
|---|---|---|---|---|---|---|
| | Agricola Bagno a Sorra | | | | | |
| **at_18** | Azienda Terzi di Monte Oliveto | Asciano (SI) | blend | | Biologic oil | 21 |
| **at_19** | Frantoio Loziro | Murlo (SI) | Frantoio, Leccino, Moraiolo | | | 22 |
| **at_20** | Azienda Agricola Carraia | Podere Carraia, Petroio (SI) | blend | 2012 | | 23 |
| **at_21** | Tenuta Montalto | San Miniato (PI) | Frantoio, Leccino, Moraiolo | 2012 | | 24 |
| **at_22** | Cosimo Maria Masina | San Miniato (PI) | Mignola | 2012 | Monocultivar | 25 |
| **at_23** | Tenuta San Quintino | San Miniato (PI) | Frantoio, Leccino, Moraiolo | 2012 | | 26 |
| **at_24** | Azienda Olearea Chianti | Greve (FI) | Frantoio, Leccino, Moraiolo | 2011 | | 27 |
| **at_25** | Azienda Agricola Donati | Casale Marittimo (PI) | Frantoio, Leccino, Moraiolo | 2012 | | 28 |
| **at_26** | Piacenza Lucia Giole | Castagneto Carducci (LI) | Frantoio, Leccino, Moraiolo | 2012 | | 29 |
| **at_27** | Villa Magra | Santa Luce (PI) | Frantoio, Leccino, Moraiolo | 2012 | | 30 |
| **at_28** | Sopra Le Vigne | Calci (PI) | Frantoio, Leccino, Moraiolo | 2012 | | 31 |
| **at_29** | Azienda Regionale Alberese | Alberese (GR) | blend | 2012 | Biologic oil | 32 |
| --------- | ------------- | ------------- | -------------------------------------------------------------- | ------- | ------------------------ | |
| **ap_1a** | Azienda Agricola Buondioli | Carpino (FG) | Frantoio | 2012 | Monocultivar | 1 |
| **ap_1b** | Azienda Agricola Buondioli | Carpino (FG) | Leccino | 2012 | Monocultivar | 2 |
| **ap_2a** | Azienda Agricola "D.Carbone" | Toritto (BA) | blend | 2010 | | 3 |
| **ap_2b** | Azienda Agricola "D.Carbone" | Toritto (BA) | blend | 2011 | | 4 |

| | | | | | | |
|---|---|---|---|---|---|---|
| **ap_2c** | Azienda Agricola "D.Carbone" | Toritto (BA) | blend | 2012 | | 5 |
| **ap_3** | Antica Azienda Agricola Ricucci | Rodi Garganco (FG) | blend | 2012 | | 6 |
| **ap_4a** | Azienda Agricola "Cuonzo Franco" | Palombaio (BA) | Ogliarola | 2012 | Monocultivar | 7 |
| **ap_4b** | Azienda Agricola "Cuonzo Franco" | Palombaio (BA) | Coratina | 2012 | Monocultivar | 8 |
| **ap_5** | Masseria Chicco Rizzo | Martignano (LE) | Cellina di Nardò, Ogliarola | 2012 | | 9 |
| **ap_6** | Cooperativa Agricola Olearia Sannicolese | Sannicola (LE) | Cellina di Nardò, Ogliarola | 2012 | | 10 |
| **ap_7** | Tenuta Agricola "Serra Cicora" | Nardò (LE) | blend | 2012 | | 11 |
| **ap_8a** | Agrié di Nicola Santoro | Cursi (LE) | Cellina di Nardò, Ogliarola | 2011 | | 12 |
| **ap_8b** | Agrié di Nicola Santoro | Cursi (LE) | Cellina di Nardò, Ogliarola | 2012 | | 13 |
| **ap_9a** | Conte | Sternatia (LE) | Coratina | 2012 | Monocultivar | 14 |
| **ap_9b** | Conte | Sternatia (LE) | Frantoio | 2012 | Monocultivar | 15 |
| **ap_9c** | Conte | Sternatia (LE) | Picholine | 2012 | Monocultivar | 16 |
| **ap_10** | Agrosì | Supersano (LE) | blend | 2012 | | 17 |
| **ap_11** | Terra del Sole | Rignano Garganico (FG) | blend | 2012 | | 18 |
| **ap_12** | Adamo | Alliste (LE) | Cellina di Nardò, Ogliarola | 2012 | | 19 |
| **ap_13a** | Agricola Nuova Generazione | Martano (LE) | blend | 2012 | Mix green-ripe olives | 20 |
| **ap_13b** | Agricola Nuova Generazione | Martano (LE) | blend | 2012 | Green olives | 21 |
| **ap_13c** | Agricola NuovaGenerazione | Martano (LE) | blend | 2012 | mature | 22 |
| **ap_14a1** | Agrinn | San Severo (FG) | blend | 2012 | silos I | 23 |

| | | | | | | |
|---|---|---|---|---|---|---|
| **ap_14a 2** | Agrinn | San Severo (FG) | Blend | 2012 | silos II | 24 |
| **ap_14a 3** | Agrinn | San Severo (FG) | blend | 2012 | silos III | 25 |
| **ap_14b 1** | Agrinn | San Severo (FG) | blend | 2012 | Biologic oil,silos 1 | 26 |
| **ap_14b 2** | Agrinn | San Severo (FG) | blend | 2012 | Biologic oil,silos 2 | 27 |
| **ap_15** | FrantoioScirop po | San Severo (FG) | Peranzana | 2012 | Monocultivar | 28 |
| **ap_16a** | Macchia del Barone | Melendugno (LE) | blend | 2012 | | 29 |
| **ap_16b** | Macchia del Barone | Melendugno (LE) | blend | 2012 | | 30 |
| **ap_17** | Bosco delleVergini | Bitonto (BA) | Cima di Bitonto | 2012 | Monocultivar | 31 |
| **ap_18** | Olio Colella | Corato (BA) | Coratina | 2012 | Monocultivar | 32 |
| **ap_19a** | Alea | Martano (LE) | Ogliarola | 2012 | Monocultivar | 33 |
| **ap_19b** | Alea | Martano (LE) | Cellina di Nardò, Ogliarola | 2012 | | 34 |
| **ap_19c** | Alea | Martano (LE) | Cellina di Nardò | 2012 | Monocultivar | 35 |

**Model parameters for Figure 2**

Monoexponential relaxation (Ms are the amplitudes in arbitrary units):

$$y = M_0 * exp\left(-\frac{t}{T_1}\right) + M_{inf}$$

$M_0 = 8.6, T_1 = 64\ ms, M_{inf} = 1.3$

Biexponential relaxation:

$$y = M_{0a} * exp\left(-\frac{t}{T_{1a}}\right) + M_{0b} * exp\left(-\frac{t}{T_{1b}}\right) + M_{inf}$$

$M_{0a} = 5.7\ , T_{1a} = 42\ ms, M_{0b} = 3.3, T_{1b} = 165\ ms, M_{inf} = 0.95$
